# 5,6-Di-2-thienyl-2,3-dihydro­pyrazine

**DOI:** 10.1107/S1600536810012705

**Published:** 2010-04-14

**Authors:** Madhukar Hemamalini, Hoong-Kun Fun

**Affiliations:** a X-ray Crystallography Unit, School of Physics, Universiti Sains Malaysia, 11800 USM, Penang, Malaysia

## Abstract

In the title compound, C_12_H_10_N_2_S_2_, which was synthesized by the reaction of 2,2′-thenil and ethyl­enediamine, the dihedral angle between the two thio­phene rings is 66.33 (9)°. In the crystal structure, inter­molecular C—H⋯N hydrogen bonds link the mol­ecules into infinite chains along the *b* axis and weak C—H⋯π inter­actions may further stabilize the structure.

## Related literature

For backgroud to thenils, see: Shimon *et al.* (1993 ▶). For related structures, see: Crundwell *et al.* (2002*a* ▶,*b* ▶, 2003 ▶); Linehan *et al.* (2003 ▶); Stacy *et al.* (2003 ▶). For bond-length data, see: Allen *et al.* (1987 ▶). For the stability of the temperature controller used in the data collection, see: Cosier & Glazer (1986 ▶).
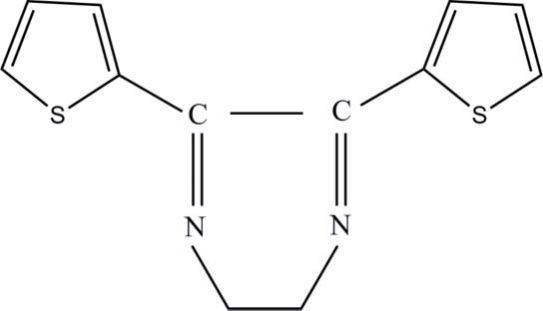


## Experimental

### 

#### Crystal data

C_12_H_10_N_2_S_2_
*M* *_r_* = 246.34
Monoclinic, 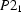

*a* = 5.5006 (9) Å
*b* = 7.5246 (12) Å
*c* = 14.116 (2) Å
β = 97.902 (5)°
*V* = 578.73 (16) Å^3^
*Z* = 2
Mo *K*α radiation
μ = 0.43 mm^−1^
*T* = 100 K
0.32 × 0.26 × 0.08 mm

#### Data collection

Bruker APEX DUO CCD area-detector diffractometer
Absorption correction: multi-scan (*SADABS*; Bruker, 2009 ▶) *T*_min_ = 0.875, *T*_max_ = 0.965
9583 measured reflections
4603 independent reflections
4295 reflections with *I* > 2σ(*I*)
*R*_int_ = 0.022

#### Refinement

*R*[*F* ^2^ > 2σ(*F* ^2^)] = 0.032
*wR*(*F* ^2^) = 0.115
*S* = 1.20
4603 reflections
145 parameters
1 restraint
All H-atom parameters refined
Δρ_max_ = 0.62 e Å^−3^
Δρ_min_ = −0.47 e Å^−3^
Absolute structure: Flack (1983 ▶), with 1899 Friedel pairs
Flack parameter: 0.07 (6)

### 

Data collection: *APEX2* (Bruker, 2009 ▶); cell refinement: *SAINT* (Bruker, 2009 ▶); data reduction: *SAINT*; program(s) used to solve structure: *SHELXTL* (Sheldrick, 2008 ▶); program(s) used to refine structure: *SHELXTL*; molecular graphics: *SHELXTL*; software used to prepare material for publication: *SHELXTL* and *PLATON* (Spek, 2009 ▶).

## Supplementary Material

Crystal structure: contains datablocks global, I. DOI: 10.1107/S1600536810012705/hb5396sup1.cif

Structure factors: contains datablocks I. DOI: 10.1107/S1600536810012705/hb5396Isup2.hkl

Additional supplementary materials:  crystallographic information; 3D view; checkCIF report

## Figures and Tables

**Table 1:** Hydrogen-bond geometry (Å, °) *Cg*2 is the centroid of the S2/C9–C12 ring.

*D*—H⋯*A*	*D*—H	H⋯*A*	*D*⋯*A*	*D*—H⋯*A*
C1—H1*A*⋯N1^i^	0.93	2.33	3.235 (3)	163
C2—H2*A*⋯*Cg*2^ii^	0.93	2.85	3.7737 (18)	171

Symmetry codes: (i) 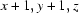; (ii) 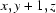.

**Fig. 1.**
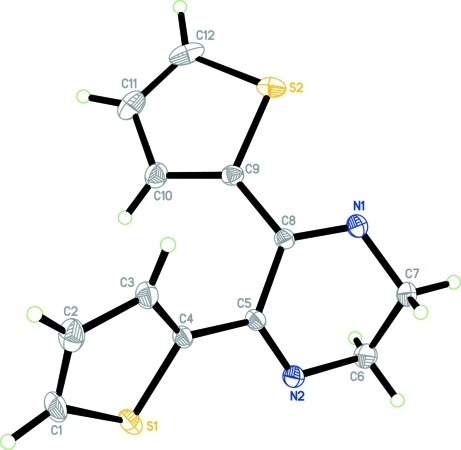
The asymmetric unit of (I) with displacement ellipsoids drawn at the 50% probability level.

**Fig. 2.**
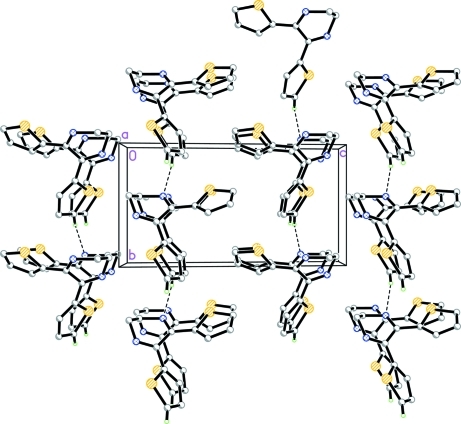
The crystal packing of (I), showing hydrogen-bonded (dashed lines) networks. H atoms are not involving the hydrogen bond interactions are omitted for clarity.

## supplementary crystallographic information

### Comment

Thienyl-based guests have shown preferential inclusion into the host by keeping thienyl ring *S* atoms pointed away from the face of growing crystals, possibly to avoid unfavorable electrostatic interactions between sulfur lone pairs coplanar with the thiophene ring and molecules already incorporated into the growing crystal face (Shimon *et al.*, 1993). The  structural studies on thenoins (Crundwell *et al.*, 2002*a*,*b*) and thenils (Crundwell *et al.*, 2003), and other thiophene-containing molecules such as 2,5-diphenyl-3,4-dithien-3-ylcyclopentadien-1-one (Linehan *et al.*, 2003) and 4-bromo-2-thiophenecarboxaldehyde (Stacy *et al.*, 2003) have been reported in the literature. In continuation of this area of study, the crystal structure of the title compound, (I), is reported here.

In the molecule of the title compound (Fig. 1), the bond lengths (Allen *et al.*, 1987) and angles are within normal ranges. The dihedral angle between the two thiophene rings S1/C1–C4 and S2/C9–C12 is 66.33 (9)°. In the crystal structure, intermolecular C—H···N hydrogen bonds (Table 1) link the molecules (Fig. 2) into infinite chains along the *b* axis, in which they may be effective in the stabilization of the structure. The crystal structure is further stabilized by C—H···π interactions (Table 1), involving the S2/C9–C12 (centroid Cg2) ring.

### Experimental

2,2'-thenil (55 mg) and ethylenediamine (15 mg) in ethanol/water (40 ml) were heated under reflux for 2 h with stirring. The resulting solution was then cooled to room temperature. After a few days of slow evaporation of the solvent, brown plates of (I) were obtained.

### Refinement

All H atoms were positioned geometrically (C—H = 0.93 or 0.97 Å) and were refined using a riding model, with *U*_iso_(H) = 1.2 or 1.5*U*_eq_(C). In the absence of significant anomalous scattering effects, 1899 Friedel pairs were merged.

### Crystal data

**Table:**

C_12_H_10_N_2_S_2_	*F*(000) = 256
*M**_r_* = 246.34	*D*_x_ = 1.414 Mg m^−^^3^
Monoclinic, *P*2_1_	Mo *K*α radiation, λ = 0.71073 Å
Hall symbol: P 2yb	Cell parameters from 5014 reflections
*a* = 5.5006 (9) Å	θ = 2.9–34.8°
*b* = 7.5246 (12) Å	µ = 0.43 mm^−^^1^
*c* = 14.116 (2) Å	*T* = 100 K
β = 97.902 (5)°	Plate, brown
*V* = 578.73 (16) Å^3^	0.32 × 0.26 × 0.08 mm
*Z* = 2	

### Data collection

**Table:**

Bruker APEX DUO CCD area-detector diffractometer	4603 independent reflections
Radiation source: fine-focus sealed tube	4295 reflections with *I* > 2σ(*I*)
graphite	*R*_int_ = 0.022
φ and ω scans	θ_max_ = 35.1°, θ_min_ = 1.5°
Absorption correction: multi-scan (*SADABS*; Bruker, 2009)	*h* = −8→8
*T*_min_ = 0.875, *T*_max_ = 0.965	*k* = −12→12
9583 measured reflections	*l* = −22→22

### Refinement

**Table:**

Refinement on *F*^2^	Secondary atom site location: difference Fourier map
Least-squares matrix: full	Hydrogen site location: inferred from neighbouring sites
*R*[*F*^2^ > 2σ(*F*^2^)] = 0.032	All H-atom parameters refined
*wR*(*F*^2^) = 0.115	*w* = 1/[σ^2^(*F*_o_^2^) + (0.0632*P*)^2^ + 0.0559*P*] where *P* = (*F*_o_^2^ + 2*F*_c_^2^)/3
*S* = 1.20	(Δ/σ)_max_ < 0.001
4603 reflections	Δρ_max_ = 0.62 e Å^−^^3^
145 parameters	Δρ_min_ = −0.47 e Å^−^^3^
1 restraint	Absolute structure: Flack (1983), with 1899 Friedel pairs
Primary atom site location: structure-invariant direct methods	Flack parameter: 0.07 (6)

### Special details

**Table:**

Experimental. The crystal was placed in the cold stream of an Oxford Cryosystems Cobra open-flow nitrogen cryostat (Cosier & Glazer, 1986) operating at 100.0 (1) K.
Geometry. All s.u.'s (except the s.u. in the dihedral angle between two l.s. planes) are estimated using the full covariance matrix. The cell s.u.'s are taken into account individually in the estimation of s.u.'s in distances, angles and torsion angles; correlations between s.u.'s in cell parameters are only used when they are defined by crystal symmetry. An approximate (isotropic) treatment of cell s.u.'s is used for estimating s.u.'s involving l.s. planes.
Refinement. Refinement of F^2^ against ALL reflections. The weighted R-factor wR and goodness of fit S are based on F^2^, conventional R-factors R are based on F, with F set to zero for negative F^2^. The threshold expression of F^2^ > 2σ(F^2^) is used only for calculating R-factors(gt) etc. and is not relevant to the choice of reflections for refinement. R-factors based on F^2^ are statistically about twice as large as those based on F, and R- factors based on ALL data will be even larger.

### Fractional atomic coordinates and isotropic or equivalent isotropic displacement parameters (Å^2^)

**Table:**

	*x*	*y*	*z*	*U*_iso_*/*U*_eq_	
S1	0.53730 (6)	0.88379 (6)	0.15567 (3)	0.02249 (9)	
S2	−0.08370 (7)	0.37500 (7)	0.39261 (3)	0.02435 (10)	
N1	−0.1511 (2)	0.42753 (19)	0.18646 (9)	0.0176 (2)	
N2	0.2016 (2)	0.58341 (19)	0.08083 (8)	0.0167 (2)	
C1	0.5372 (3)	1.0745 (3)	0.22117 (12)	0.0256 (3)	
H1A	0.6518	1.1651	0.2206	0.031*	
C2	0.3468 (3)	1.0777 (2)	0.27490 (12)	0.0225 (3)	
H2A	0.3166	1.1719	0.3143	0.027*	
C3	0.2025 (3)	0.9222 (2)	0.26367 (10)	0.0176 (2)	
H3A	0.0665	0.9026	0.2948	0.021*	
C4	0.2854 (2)	0.8021 (2)	0.20133 (9)	0.0141 (2)	
C5	0.1765 (2)	0.6334 (2)	0.16622 (10)	0.0141 (2)	
C6	0.0602 (3)	0.4259 (2)	0.04601 (10)	0.0199 (3)	
H6A	0.1494	0.3194	0.0686	0.024*	
H6B	0.0373	0.4245	−0.0234	0.024*	
C7	−0.1875 (3)	0.4280 (2)	0.08163 (10)	0.0192 (3)	
H7A	−0.2783	0.5333	0.0582	0.023*	
H7B	−0.2817	0.3244	0.0579	0.023*	
C8	0.0286 (2)	0.5231 (2)	0.22635 (9)	0.0136 (2)	
C9	0.0980 (3)	0.5061 (2)	0.32993 (9)	0.0148 (2)	
C10	0.3080 (3)	0.5582 (2)	0.38767 (10)	0.0199 (3)	
H10A	0.4286	0.6292	0.3670	0.024*	
C11	0.3203 (4)	0.4914 (3)	0.48252 (11)	0.0272 (3)	
H11A	0.4496	0.5144	0.5307	0.033*	
C12	0.1220 (4)	0.3904 (3)	0.49497 (11)	0.0296 (4)	
H12A	0.1007	0.3360	0.5524	0.036*	

### Atomic displacement parameters (Å^2^)

**Table:**

	*U*^11^	*U*^22^	*U*^33^	*U*^12^	*U*^13^	*U*^23^
S1	0.02124 (15)	0.02353 (19)	0.02375 (16)	−0.01080 (15)	0.00690 (12)	−0.00227 (15)
S2	0.02570 (17)	0.0293 (2)	0.01976 (15)	−0.00416 (17)	0.00917 (12)	0.00490 (16)
N1	0.0195 (5)	0.0164 (5)	0.0168 (4)	−0.0061 (4)	0.0022 (4)	−0.0014 (4)
N2	0.0188 (5)	0.0166 (5)	0.0151 (5)	−0.0038 (4)	0.0037 (4)	−0.0023 (4)
C1	0.0311 (8)	0.0176 (7)	0.0264 (7)	−0.0130 (6)	−0.0022 (6)	0.0022 (6)
C2	0.0283 (7)	0.0127 (6)	0.0246 (6)	−0.0012 (6)	−0.0030 (5)	−0.0006 (5)
C3	0.0178 (5)	0.0161 (6)	0.0186 (5)	−0.0030 (5)	0.0010 (4)	−0.0018 (5)
C4	0.0142 (5)	0.0126 (6)	0.0151 (5)	−0.0027 (4)	0.0007 (4)	0.0008 (4)
C5	0.0138 (5)	0.0131 (6)	0.0154 (5)	−0.0023 (4)	0.0019 (4)	0.0006 (4)
C6	0.0256 (6)	0.0162 (6)	0.0181 (5)	−0.0039 (5)	0.0042 (5)	−0.0054 (5)
C7	0.0203 (6)	0.0200 (7)	0.0164 (5)	−0.0066 (5)	0.0001 (4)	−0.0024 (5)
C8	0.0154 (5)	0.0113 (5)	0.0141 (5)	−0.0022 (4)	0.0027 (4)	0.0000 (4)
C9	0.0180 (5)	0.0129 (6)	0.0140 (5)	−0.0001 (5)	0.0036 (4)	0.0004 (4)
C10	0.0205 (6)	0.0206 (7)	0.0173 (5)	−0.0006 (5)	−0.0017 (5)	0.0012 (5)
C11	0.0331 (8)	0.0304 (9)	0.0163 (6)	0.0034 (7)	−0.0030 (5)	0.0006 (6)
C12	0.0399 (8)	0.0353 (10)	0.0151 (5)	0.0070 (9)	0.0087 (5)	0.0054 (7)

### Geometric parameters (Å, °)

**Table:**

S1—C1	1.707 (2)	C4—C5	1.461 (2)
S1—C4	1.7201 (14)	C5—C8	1.5040 (19)
S2—C12	1.7124 (19)	C6—C7	1.516 (2)
S2—C9	1.7317 (14)	C6—H6A	0.9700
N1—C8	1.2874 (18)	C6—H6B	0.9700
N1—C7	1.4658 (19)	C7—H7A	0.9700
N2—C5	1.2880 (18)	C7—H7B	0.9700
N2—C6	1.465 (2)	C8—C9	1.4652 (19)
C1—C2	1.375 (3)	C9—C10	1.376 (2)
C1—H1A	0.9300	C10—C11	1.423 (2)
C2—C3	1.411 (2)	C10—H10A	0.9300
C2—H2A	0.9300	C11—C12	1.361 (3)
C3—C4	1.382 (2)	C11—H11A	0.9300
C3—H3A	0.9300	C12—H12A	0.9300
			
C1—S1—C4	92.13 (8)	C7—C6—H6B	109.7
C12—S2—C9	91.82 (8)	H6A—C6—H6B	108.2
C8—N1—C7	115.50 (12)	N1—C7—C6	109.29 (12)
C5—N2—C6	115.48 (12)	N1—C7—H7A	109.8
C2—C1—S1	111.76 (12)	C6—C7—H7A	109.8
C2—C1—H1A	124.1	N1—C7—H7B	109.8
S1—C1—H1A	124.1	C6—C7—H7B	109.8
C1—C2—C3	112.51 (15)	H7A—C7—H7B	108.3
C1—C2—H2A	123.7	N1—C8—C9	117.84 (12)
C3—C2—H2A	123.7	N1—C8—C5	120.25 (12)
C4—C3—C2	112.59 (13)	C9—C8—C5	121.61 (12)
C4—C3—H3A	123.7	C10—C9—C8	130.47 (13)
C2—C3—H3A	123.7	C10—C9—S2	110.85 (10)
C3—C4—C5	128.94 (12)	C8—C9—S2	118.02 (10)
C3—C4—S1	110.99 (11)	C9—C10—C11	112.61 (15)
C5—C4—S1	119.83 (10)	C9—C10—H10A	123.7
N2—C5—C4	118.67 (13)	C11—C10—H10A	123.7
N2—C5—C8	120.18 (13)	C12—C11—C10	112.55 (15)
C4—C5—C8	121.10 (11)	C12—C11—H11A	123.7
N2—C6—C7	109.89 (12)	C10—C11—H11A	123.7
N2—C6—H6A	109.7	C11—C12—S2	112.16 (12)
C7—C6—H6A	109.7	C11—C12—H12A	123.9
N2—C6—H6B	109.7	S2—C12—H12A	123.9
			
C4—S1—C1—C2	−1.31 (14)	C7—N1—C8—C5	3.3 (2)
S1—C1—C2—C3	0.95 (19)	N2—C5—C8—N1	−29.3 (2)
C1—C2—C3—C4	0.1 (2)	C4—C5—C8—N1	148.07 (15)
C2—C3—C4—C5	−175.19 (14)	N2—C5—C8—C9	144.36 (15)
C2—C3—C4—S1	−1.03 (16)	C4—C5—C8—C9	−38.3 (2)
C1—S1—C4—C3	1.33 (12)	N1—C8—C9—C10	163.90 (16)
C1—S1—C4—C5	176.10 (12)	C5—C8—C9—C10	−9.9 (2)
C6—N2—C5—C4	−172.06 (13)	N1—C8—C9—S2	−5.87 (19)
C6—N2—C5—C8	5.4 (2)	C5—C8—C9—S2	−179.66 (11)
C3—C4—C5—N2	147.03 (16)	C12—S2—C9—C10	−0.12 (14)
S1—C4—C5—N2	−26.69 (19)	C12—S2—C9—C8	171.56 (13)
C3—C4—C5—C8	−30.4 (2)	C8—C9—C10—C11	−170.37 (16)
S1—C4—C5—C8	155.92 (11)	S2—C9—C10—C11	−0.03 (18)
C5—N2—C6—C7	37.17 (19)	C9—C10—C11—C12	0.2 (2)
C8—N1—C7—C6	38.97 (19)	C10—C11—C12—S2	−0.3 (2)
N2—C6—C7—N1	−60.13 (17)	C9—S2—C12—C11	0.26 (17)
C7—N1—C8—C9	−170.61 (13)		

### Hydrogen-bond geometry (Å, °)

**Table:**

*D*—H···*A*	*D*—H	H···*A*	*D*···*A*	*D*—H···*A*
C1—H1A···N1^i^	0.93	2.33	3.235 (3)	163
C2—H2A···Cg2^ii^	0.93	2.85	3.7737 (18)	171

Symmetry codes: (i) *x*+1, *y*+1, *z*; (ii) *x*, *y*+1, *z*.
